# The Rice NAD^+^-Dependent Histone Deacetylase OsSRT1 Targets Preferentially to Stress- and Metabolism-Related Genes and Transposable Elements

**DOI:** 10.1371/journal.pone.0066807

**Published:** 2013-06-25

**Authors:** Xiaochao Zhong, Hua Zhang, Yu Zhao, Qianwen Sun, Yongfeng Hu, Hai Peng, Dao-Xiu Zhou

**Affiliations:** 1 National Key Laboratory of Crop Genetic Improvement, Huazhong Agricultural University, Wuhan, China; 2 Institut de Biologie des Plantes, Université Paris-sud 11, Orsay, France; 3 Institute of Interdisciplinary Science Research Institute, Jianghan University, Wuhan, China; University of Delhi South Campus, India

## Abstract

Histone acetylation/deacetylation is an important chromatin modification for epigenetic regulation of gene expression. Silent information regulation2 (Sir2)-related sirtuins are nicotinamide-adenine dinucleotide (NAD^+^)-dependent histone deacetylases (HDAC). The mammalian sirtuin family comprises 7 members (SIRT1-7) that act in different cellular compartments to regulate metabolism and aging. The rice genome contains only two Sir2-related genes: OsSRT1 (or SRT701) and OsSRT2 (orSRT702). OsSRT1 is closely related to the mammalian SIRT6, while OsSRT2 is homologous to SIRT4. Previous work has shown that OsSRT1 is required for the safeguard against genome instability and cell damage in rice plant. In this work we investigated the role of OsSRT1 on genome-wide acetylation of histone H3 lysine 9 (H3K9ac) and studied the genome-wide binding targets of OsSRT1. The study reveals that OsSRT1 binds to loci with relatively low levels of H3K9ac and directly regulates H3K9ac and expression of many genes that are related to stress and metabolism, indicating that OsSRT1 is an important site-specific histone deacetylase for gene regulation in rice. In addition, OsSRT1 is found to also target to several families of transposable elements, suggesting that OsSRT1 is directly involved in transposable element repression.

## Introduction

Histone lysine acetylation is an important epigenetic modification for genome function and gene activity. Dynamic modulation of histone acetylation in plants has been shown to be involved in gene expression programs of plant development and responses to environmental conditions [1], [2]. Histone acetylation homeostasis is regulated by the antagonistic actions of histone acetyltransferases (HAT) and histone deacetylases (HDAC). The rice genome contains at least 19 HDAC genes belonging to three classes [3]. Among them, two classes are conserved with the yeast RPD3 (Reduce Potassium Dependency 3) and SIR2 (Silent Information Regulation2) HDACs. The third group, known as HD2, is only found in plants. Yeast SIR2 is a NAD^+^-dependent HDAC required for transcriptional gene silencing of the mating type loci, telomeres and ribosomal repeats. Homologs of the SIR2 protein are called sirtuins and have been implicated in a variety of processes including transcriptional repression, metabolism, apoptosis, and aging in animal cells [4]. In human cells there are 7 sirtuins (SIRT1-7) that have different subcellular localizations. SIRT1 is predominantly localized in the nucleus but also functions in the cytoplasm, while SIRT2 is essentially found in the cytoplasm. SIRT6 and SIRT7 are nuclear, while SIRT3, SIRT4 and SIRT5 are mitochondrial proteins [4]. Among the sirtuins, SIRT6 has been shown to deacetylate H3K9 and H3K56 and to play critical roles in gene expression programs related to aging and metabolic homeostasis and in preventing genome instability in animal cells [5].

Plant genomes contain only two sirtuin-like genes. One is closely related to SIRT6, the other to SIRT4 [3], [6], [7]. The rice homolog of SIRT6 (OsSRT1 also called SRT701) is localized in the nucleus, while that of SIRT4 (OsSRT2 or SRT702) is found in mitochondria [6], [8]. Down-regulation of *OsSRT1* by RNAi produces a lesion-mimic phenotype and induces H3K9 acetylation (H3K9ac) and expression of hypersensitive response-related genes in rice plants [6]. In addition, the transcription of many transposable elements is activated in the RNAi plants, indicating that transposons and cell death-related genes might be among the primary targets of OsSRT1-mediated gene silencing.

The homeostasis of histone H3K9 acetylation regulated by OsSRT1 in controlling genome-wide gene expression remained unclear. It was not known whether OsSRT1 directly targeted to specific genes to undergo histone deacetylation. To study the chromatin function and to identify direct binding targets of the OsSRT1, we first compared the genome-wide H3K9 acetylation profiles between wild type and OsSRT1 down-regulation plants, and then investigated the genomic binding sites of OsSRT1 by chromatin immunoprecipitation assays. Our data indicated that H3K9ac was mostly associated with active genes in rice. *OsSRT1* down-regulation did not drastically alter the genome-wide H3K9ac landscape, but led to increases of H3K9ac over a subset of genes belonging to specific functional categories and transposable elements. OsSRT1 was found to be associated with 1824 genes that showed relatively low levels of H3K9ac. In addition, OsSRT1 binding was significantly enriched in the gene bodies compared to the upstream and the down regions. Gene ontology analysis revealed that OsSRT1 preferentially targets to genes involved in stress-responses and metabolisms and to several families of transposable elements in the rice genome, indicating that OsSRT1 is a site-specific HDAC involved in specific gene regulation and transposon repression.

## Materials and Methods

### Plant Materials

Rice (*Oryza sativa*) cultivar Minghui 63 (MH63) was used in this study. The *OsSRT1* RNAi line was previously reported by Huang et al. (2007) [6].

### Chromatin Immunoprecipitation (ChIP)

ChIP experiments were performed as described [6]. Rice seedlings were grown under 14 h light/10 h dark cycles at 25°C–28°C in ½ Murashige and Skoog media. Two grams of 11-day old seedlings were harvested and cross linked in 1% formaldehyde under vacuum. Chromatin was extracted and fragmented to 200–500 bp by sonication and ChIP was performed by using two antibodies: anti-H3K9ac or anti-OsSRT1. Anti-H3K9ac was purchased from Millipore (07–352). Anti-OsSRT1 was prepared by immunizing rabbits with *E. coli*-produced OsSRT1 protein. The anti-serum was affinity purified with protein-A agarose beads purchased from Millipore (16–157) and tested by Western blots to detect the OsSRT1 protein in rice tissues (Fig. S1). The precipitated and input DNAs were then analyzed by real-time PCR with gene-specific primers. Real-time PCR was performed in a total volume of 25 µL with 1.0 µL of the ChIP products, 0.25 µM primers, and 12.5 µL SYBR Green Master mix (TAKARA) on a 7500 real-time PCR machine (Applied Biosystems) according to the manufacturer's instructions. All primers were annealed at 60°C and run for 40 cycles for ChIP products. The ChIP enrichment for H3K9ac or OsSRT1-binding was quantified by comparing the threshold cycle (C_t_) of the ChIP sample with that of the input with: 2^(Ct of input-Ct of sample ChIP)^ [9]. Three technical repeats were performed.

### ChIP-seq

Precipitated DNA from ChIP assays was PCR amplified with Illumina primers and library fragments of 100–300 bp (insert plus adaptor and PCR primer sequences) were isolated from agarose gels. The purified DNA was captured on an Illumina flow cell for cluster generation. Libraries were sequenced with Illumina Genome Analysis.

### Data Analysis

Sequencing reads from all libraries were mapped to the reference genome (MSU6.0) using MAQ software with default parameters [10]. Reads which could be mapped equally well to unique locations without mismatch or with less than two mismatches were retained for further analysis. Genomic regions associated with histone H3K9ac or OsSRT1-binding were identified using MACS software [11], in which default parameters (bandwidth, 300 bp; mfold, 32; *p*-value of 1.00e-05) and largellocal = 5000 were set up to call peaks representing enriched H3K9Ac or OsSRT1 binding. Peaks in the gene body and the upstream and downstream 2 Kb regions were identified as histone H3K9ac or OsSRT1 binding. The transcription factors in the genome were classified according to PlantTFDB (http://planttfdb.cbi.edu.cn/index.php?sp=Osj).

Gene ontology classification provided in TIGR Rice Genome Annotation Project (http://rice.plantbiology.msu.edu/) was used to assign genes to a hierarchical biological process following the criteria of the Gene Ontology Consortium databases (http://www.geneontology.org/external2go/tigr2go), then the Web Gene Ontology Annotation Plotting tool WEGO was used to plot GO categories (http://wego.genomics.org.cn) [12]. The *p*-value of a particular pathway was calculated with Pearson’s chi-squared test, then corrected by False discovery rate (FDR), with a FDR cutoff of 0.05 as the significance threshold [13]. The ChIP-seq data from this publication is submitted to the GEO database (accession number GSE40054). The accession number of *OsSRT1* RNAi microarray is GSE71976.

To study the distribution of transposons in the genome, we used Repeatmaske (with RM-BLAST as searching engine, the rice transposon sequence database was downloaded in Repbase (http://www.girinst.org/repbase/) to analyze the MSU6.0 version of the rice genomic sequence in high sensitivity. A transposon is considered to have H3K9 acetylation if located inside a peak. We used 2-fold cut-off in analyzing H3K9 acetylation. For TEs numbers less than 100, Fisher’s exact tests were used to test the enrichment. Hyper geometric distribution tests were used for TE numbers greater than 100. We set the *p*-value cut-off as 0.01.

We used MEME (mod anr -nmotifs 5 -minw 5 -maxw 50) to analyze the binding motif of OsSRT1 ChIP-seq peak sequences. MAST software was used to analyze the distribution of the motifs in the MSU6.0 rice genome. Fisher exact test was used in testing enrichment significance.

## Results

### Effect of *OsSRT1* RNAi on Genome-wide Histone H3K9 Acetylation in Rice

**Figure 1:**
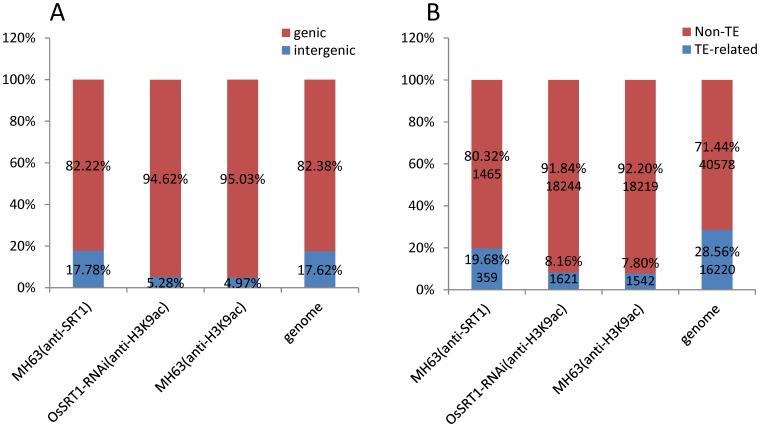
Distribution of H3K9ac and OsSRT1 ChIP-seq read peaks in the genome. A. Distribution of OsSRT1 ChIP-seq (in wild type MH63 plants) and H3K9ac ChIP-seq (in OsSRT1 RNAi and wild type MH63 plants) read peaks in genic versus intergenic regions. Genic region contains the gene body and 2Kb upstream/downstream regions. B. Distribution of ChIP-seq read peaks in transposable element (TE)-related genes versus non-TE genes.

**Figure 2:**
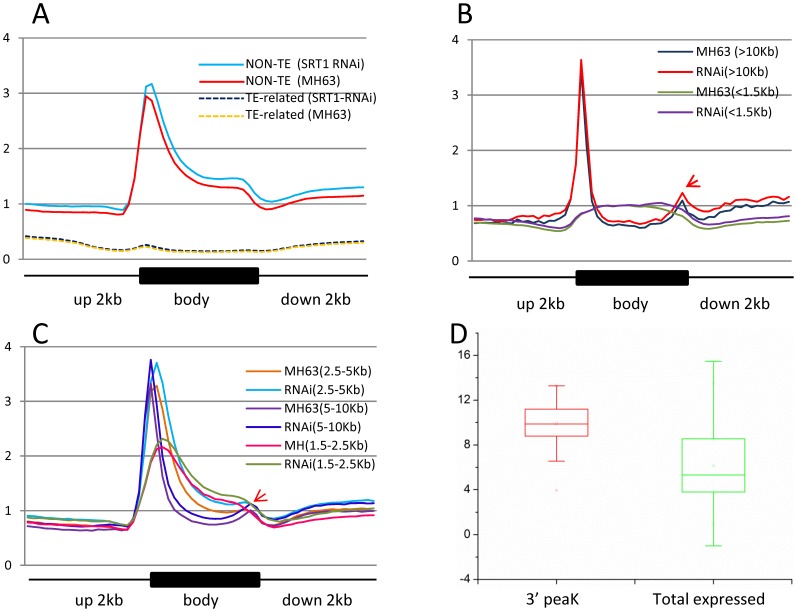
Distribution of H3K9ac over genes. A. H3K9ac distribution is enriched at transcriptional start sites (TSS) of non-TE related genes. Compared to wild type (MH63), OsSRT1 RNAi enhanced the enrichment of H3K9ac at TSS of marked genes. B. C. The TSS enrichment of H3K9ac is detected over genes larger than 1.5 kb. A 3′ end peak of H3K9ac is indicated by arrows. D. Genes with the 3′ peak of H3K9ac show higher expression levels than the average of total expressed genes. *Y* axis in A–C: tag density. The upstream, gene body and downstream regions were divided each into 20 intervals. Tag density = tag number divided by the length of the intervals. *y* axis in D: microarray signals (log2).

**Figure 3:**
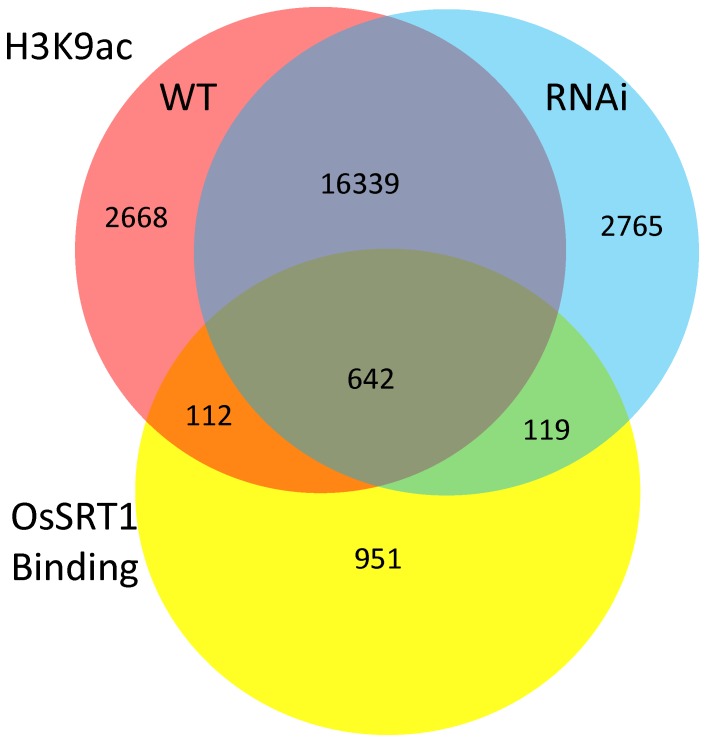
Effect of OsSRT1 RNAi on H3K9ac. Venn diagram of number of genes marked by H3K9ac in wild type (red) and in *OsSRT1* RNAi (blue) plants, and the overlapping with OsSRT1-binding genes (yellow).

**Figure 4:**
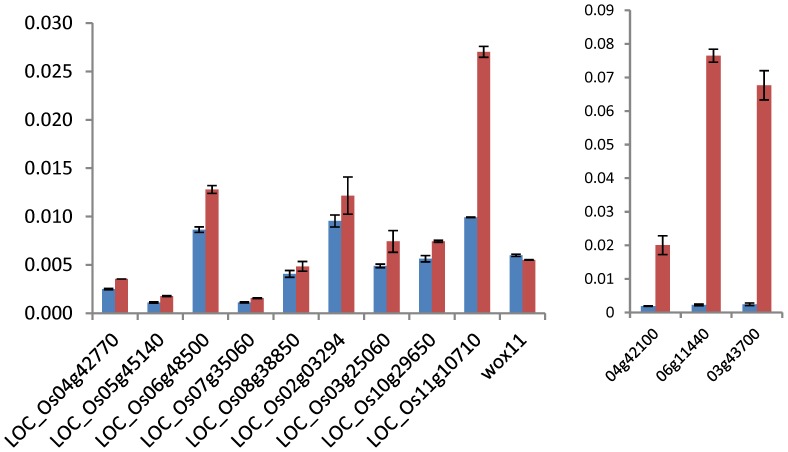
ChIP-PCR tests of H3K9ac on 12 genes in wild type and RNAi plants. Red bars represent the wild type and blue bars represent the RNAi plants. Nine non-TE (left) and 3 TE-related (right) genes were randomly selected. OsWOX11 on which H3K9ac was not detected in the ChIP-seq data was used as a negative control. *y* axis: values relative to input signals.

To study the effect of *OsSRT1* down-regulation on histone acetylation in rice, a high-resolution map of the genome-wide distribution of H3K9ac in 11-day old wild type and *OsSRT1* RNAi seedlings was determined by chromatin immunoprecipitation (ChIP) assays followed by high-throughput Illumina/Solexa sequencing (Fig. S1). Values of normalized strand correlation (NSC) and relative strand correlation (RSC) indicated a high quality of the sequence data (Fig. S2). Sequencing reads were mapped to the reference genome of rice (*Oryza sativa* L. ssp. *Japonica* cv. Nipponbare) using MAQ software [10]. About 8–9 million unique sequence reads per sample were identified and mapped to the reference genome (Fig. S2). Genomic regions or peaks associated with H3K9ac were identified using MACS 1.4 software [11]. About 95% of the peaks were mapped to the genic regions which include the gene bodies plus 2 kb upstream and 2 kb downstream regions (Fig. 1A). H3K9ac was found in about 45% (18219/40578) of the rice non-transposable element (TE)-related genes, compared to less than 10% (1542/16220) of TE-related genes in both the RNAi and wild type plants (Fig. 1B). H3K9ac was found to be clearly enriched near the 5′ transcriptional start site (TSS) of non-TE-related genes (Fig. 2A). The enrichment at the 5′ end was more pronounced for genes larger than 1.5 kb (Fig. 2B, 2C). In smaller genes (<1.5 kb), there was no such a clear enrichment (Fig. 2B). An enhanced enrichment in genes with sizes between 1.5 and 10 Kb was observed in the RNAi plants (Fig. 2C). In addition, a small peak of H3K9ac was observed at the 3′ end of the bodies of large genes (>5 kb) (Fig. 2B, 2C). Although the function of the 3′ end peak of H3K9ac was not known, these genes showed higher than average gene expression levels (Fig. 2D). One possible explanation was that the 3′ peak might be due to enriched H3.3 over active large genes in plants [14]. A total of 19671 genes were found to be marked by H3K9ac in wild type seedlings (Fig. 3), more than 91% of which overlapped with previously published genes that are marked by H3K9ac in rice seedlings [15]. Comparison of the H3K9ac ChIP-seq data with the 11 day-old rice seedling transcriptomic data corroborated the correlation between H3K9ac and gene expression (). A similar number of H3K9ac-marked genes were found in *OsSRT1* RNAi plants, of which 93% were overlapped with those from the wild type plants (Fig. 3). A small fraction of genes gained or lost H3K9ac. Likely, *OsSRT1* RNAi did not drastically alter the genome-wide H3K9ac landscape. Increases of H3K9ac on 12 genes (9 non-TE and 3 TE-related) were confirmed by ChIP-PCR (Fig. 4). Gene ontology analysis of the genes with >2-fold H3K9ac increases revealed a clear enrichment (FDR<0.05) of those involved in metabolism, transcription and response to stimulus (Fig. S4), suggesting that OsSRT1 may regulate H3K9ac levels over these specific classes of genes.

### Identification of Genome-wide OsSRT1-binding Targets

**Figure 5:**
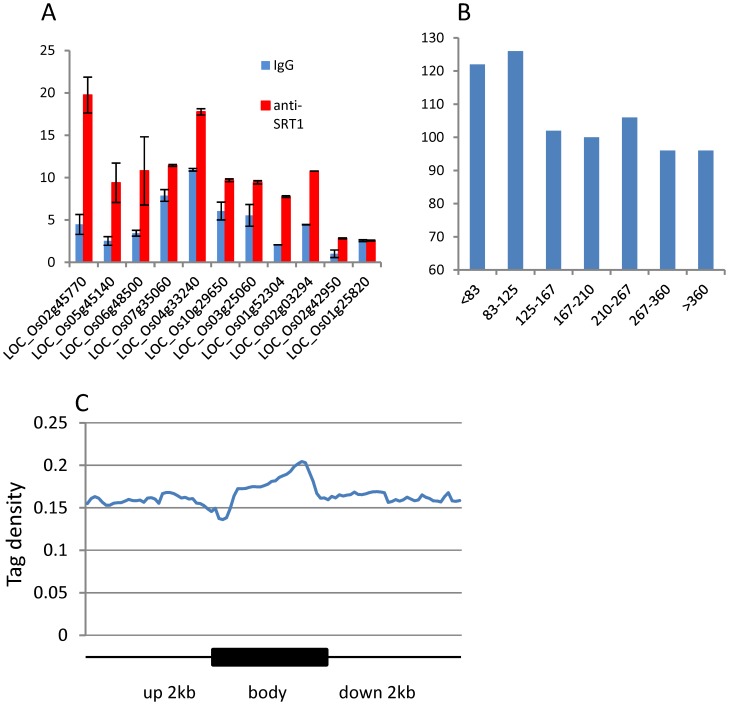
Identification of OsSRT1-binding targets. A. OsSRT1 ChIP-PCR tests of 10 genes randomly selected from the ChIP-seq gene list. IgG-ChIP was used as control. Os01g25820 that was not in the OsSRT1-binding gene list was used as a negative control. B. A higher number of OsSRT1-binding genes showed lower levels of H3K9ac. H3K9ac-marked genes were divided into 7 intervals according ChIP-seq read numbers (*x* axis), which were compared to the OsSRT1 ChIP data (*y* axis). D. Distribution of OsSRT1-binding over the target gene bodies and 2 kb upstream and downstream regions. Each gene was divided into 20 intervals and the upstream and downstream region into 100 bp intervals. The *y* axis is the average read coverage of intervals divided by total reads and by the length of the intervals.

To study the genome-wide binding sites of OsSRT1, ChIP-seq experiments with anti-OsSRT1 was performed. A total of 15.24 million reads that were analyzed for NSC and RSC for quality check (Fig. S2), were mapped to the genome. Peaks of sequence reads were identified by using MACS 1.4. About 82% of the peaks were mapped to the genic regions (the gene bodies plus 2 kb upstream and 2 kb downstream regions) (Fig. 1A). OsSRT1 was found to be associated with 1824 genes (1465 non-TE related and 259 TE-related genes) (Fig. 1B). ChIP-PCR tests of 10 of the genes confirmed the ChIP-seq data (Fig. 5A). Comparison of the OsSRT1 and H3K9ac ChIP-seq peaks revealed that about 12.5% of the OsSRT1-binding peaks overlapped with that of H3K9ac. Therefore, in most cases OsSRT1 was not recruited to H3K9ac-enriched loci. In fact, OsSRT1 tended to bind to genes with relatively low levels of H3K9ac (Fig. 5B). Interestingly, the OsSRT1 binding was significantly enriched (*p*-value = 2.78E-09) in the gene bodies compared to the upstream or down regions, with a peak toward the 3′ end of the gene bodies (Fig. 5C). Gene ontology analysis revealed also a clear enrichment (FDR<0.05) of OsSRT1-associated genes in metabolism, transcription and response to stimulus (Fig. S5). To refine this analysis, we analyzed the transcription factor gene family. A significant number (*p*-value<0.05) of transcription factor genes were found to show OsSRT1-binding and increased H3K9ac in *OsSRT1* RNAi plants (Table 1).

**Table 1:** Enrichment of transcription factor (TF) genes for OsSRT1-binding.

	TF genes	*P-*value(F)	Total genes
H3K9ac increased in RNAiplants	182	9.4e-11*	3688
SRT1-binding	73	0.013*	1824
SRT1-binding and with H3K9ac	34	0.016*	755
SRT1-binding and with H3k9ac increase in RNAi plants	9	0.0526	157
Genome-wide	1728		56798

* significance of enrichments with *p*-value<0.05 by Fisher exact tests (F).

**Figure 6:**
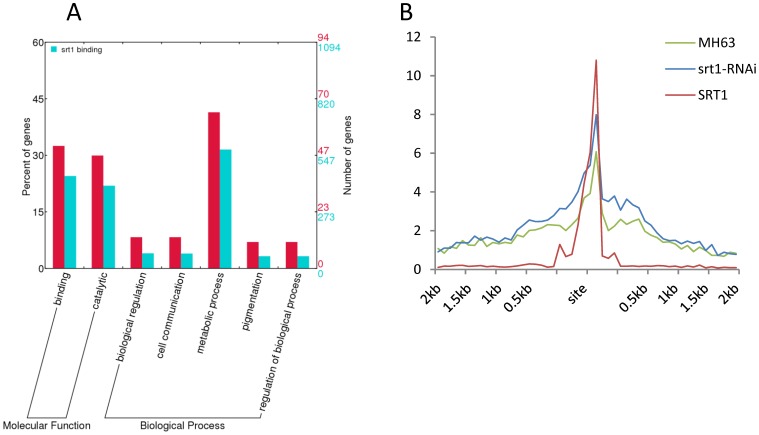
Genes that showed both OsSRT1-binding and H3K9ac increase in RNAi plants. A. 157 genes that showed both OsSRT1-binding and increased H3K9ac in *OsSRT1* RNAi plants (Red) were more enriched for metabolic pathway than the total OsSRT1-binding genes (Blue). B. Overlapping of OsSRT1-binding with increased H3K9ac in RNAi versus wild type plants in 37 of the 157 genes. *Y* axis: tag density defined as in Figures 2 and 5.

In total there were 157 genes with both OsSRT1-binding and >2-fold increases of H3K9ac in the RNAi plants (Table S1). These genes might be potentially direct targets of OsSRT1 for H3K9 deacetylation. Some of the genes were up-regulated in the RNAi plants as revealed by microarray assays (Table S1). Gene ontology analysis revealed that these genes were more enriched for metabolic pathways compared to the total OsSRT1-binding genes (Fig. 6A). Thirty seven genes showed both the H3K9ac increase in the RNAi plants and the OsSRT1-binding (Fig. 6B; Fig. S6), suggesting that OsSRT1 directly targets to these genes for H3K9 deacetylation.

### OsSRT1-binding is Enriched Over Specific Families of Transposable Elements

Previous studies showed that *OsSRT1* down-regulation leads to derepression of many transposable elements in rice [6]. Here we analyzed whether transposable element chromatin was modified by H3K9 acetylation or targeted by OsSRT1. H3K9ac was found to be enriched over the long Interspersed Nuclear Elements (LINE) and several DNA element families (i.e. hAT, MITE and Mutator) (*p*-value<0.01), but not found over the other retroelement families (Table 2, Table 3). OsSRT1-binding was found to be enriched over several families of the retroelements (i.e. Gypsy, LTR and LINE families) and the Mutator family of the DNA elements (*p*-value<0.01) (Table 2, Table 3). These data were consistent with the derepression of a large number of genes and transposable elements in *OsSRT1* RNAi plants [6], indicating that OsSRT1 may play an important role for the repression of specific families of transposable elements.

**Table 2:** Histone H3K9 acetylation and OsSRT1-binding on retrotransposons.

	Copia	Gypsy	Total LTR	LINE	SINE	Total Retro-elements
H3K9ac in WT	222(1)	690(1)	974(1)	289(0.154)	378(0.0226)	1641(1)
H3K9ac in RNAi	232(1)	739(1)	1032(1)	316*(3.36E-04)	312(0.810)	1660(1)
H3K9ac increasein RNAi	89(1)	277(1)	383(1)	126*(2.70E-04)	97(0.638)	606(1)
SRT1-binding(Fisher)	50(0.0899)	287*(0)	350*(0)	44*(1.18E-05)	22(0.0864)	416*(7.67E-12)
Genome-wide	12562	54330	69998	6627	8304	84929

In parentheses are indicated *p*-values calculated with Hypergeometric distribution tests or with Fischer exact tests as indicated.

* Significance of enrichment with *p*-value<0.01.

**Table 3:** Histone H3K9 acetylation and OsSRT1-binding on DNA transposable elements.

	hAT	En-Spm	MITE	Mutator	Helitron	Unclassified	Total DNA elements
H3K9ac in WT	497*(3.27E-03)	746(1)	5720*(0)	3000*(0)	220(1)	618(0.652)	10801*(0)
H3K9ac in RNAi	486*(8.38E-04)	737(1)	5480*(0)	2722*(0)	217(1)	616(0.260)	10258*(0)
H3K9ac increase in RNAi	162(0.124)	286(1)	2043*(1.88E-10)	739*(1.67E-11)	89(1)	237(0.0314)	3556*(2.88E-97)
SRT1-binding (Fisher)	29(0.869)	111(0.0623)	220(1)	166*(3.01E-03)	23(0.997)	20(1)	569(1)
Genome-wide	10714	29433	108347	41408	11766	15233	216901

In parentheses are indicated *p*-values calculated with Hypergeometric distribution tests or with Fischer exact tests as indicated. *Significance of enrichment with *p*-value<0.01.

### Identification of Potential OsSRT1-binding DNA Sequences

To find potential DNA motifs associated with OsSRT1, we analyzed OsSRT1-binding peaks by using MEME software, a sequence of 31 bp was found in 28 peaks. This sequence contained an inverted repeat motif with a highly conserved half motif (5′ GATGGGCCGA) that was found within the LTR of the Gypsy retrotransposons. In the rice genome we found that 1209 genes (with 2 kb up- and downstream regions, *p*-value<1E-06) contained the inverted repeats. The motif was significantly enriched among OsSRT1-binding genes (55/1824, *p*-value = 0.0062), suggesting that OsSRT1 may target to this DNA sequence.

## Discussion

H3K9ac is an important epigenetic mark associated with gene activation. The regulation of H3K9ac homeostasis is not fully understood in plants, although histone acetyltransferases targeting to H3K9 have been identified [16]. The analysis of genome-wide H3K9ac in *OsSRT1* RNAi plants indicates that OsSRT1 is a site-specific deacetylase involved in H3K9 deacetylation over many specific genes and TEs. The observations that H3K9ac was enriched at the 5′ end of genes larger than 1.5 kb and *OsSRT1* down-regulation enhanced the enrichment at that position (Fig. 2B, C), suggest that transcriptional start sites may be targeted by OsSRT1. However, the OsSRT1-binding was found to be enriched over the gene bodies. While the meaning of this binding profile is not yet clear, it is speculated that OsSRT1 may contribute to maintain a low level of H3K9ac in gene bodies. In addition, this work revealed that about 45% of rice genes are marked by H3K9ac in 11 day-old seedlings. This percentage corresponds to that of genes marked by H3K4me3 surveyed from seedlings at the same development stage [17]. By contrast, H3K27me3, a gene repression marker, is deposited over a much smaller number of genes in the rice genome [17]. Comparison of H3K9ac- and H3K4me3-marked genes revealed that 76% to 92% of H3K9 acetylated genes were also marked by H3K4me3 [15], [17], indicating that the two modifications co-mark active genes in rice.

The enrichment of genes involved in stimulus (stress) responses for OsSRT1-binding is consistent with the lesion mimic phenotypes observed in *OsSRT1* down-regulation plants and the enhanced tolerance to oxidative stress in *OsSRT1* over-expression plants [6]. Hypersensitive response-related genes were activated in the RNAi plants [6]. Possibly, OsSRT1 may regulate genes involved in stimulus-responses by H3K9 deacteylation, as this category of genes showed increased H3K9ac in *OsSRT1* RNAi plants. In addition, the finding that metabolism-related genes displayed both increased H3K9ac in *OsSRT1* RNAi plants and OsSRT1-binding suggests that OsSRT1 is preferentially involved in metabolic regulation in plants. As NAD^+^-dependent HDACs, sirtuins including OsSRT1 are biochemically linked to cellular metabolisms because NAD^+^ levels influence cellular redox and energy states. In animal cells several sirtuins function as crucial regulators of the networks that control glucose and fat metabolism in response to physiological changes in energy levels. Human SIRT6 is shown to interact physically with HIFα, a transcriptional regulator of glucose metabolism genes, and associate with the promoters of HIFα target genes in a HIFα-dependent manner and regulate histone acetylation levels on these promoters [18]. Considering the specificity of plant cells where primary metabolism and energy states are tightly linked to photosynthesis and photorespiration, it will be of interest to show whether and how OsSRT1 regulates plant energy homeostasis.

Yeast SIR2 and mammalian SIRT6 proteins are shown to be involved in telomere stability or silencing [19], [20]. However, no OsSRT1 ChIP-seq read contained the rice telomere sequence repeats, suggesting that OsSRT1 may not target to telomeres. By contrast, several lines of evidence suggest that OsSRT1 is involved in silencing of transposable elements in rice. First, about 18% of OsSRT1-bindig targets were localized to the non genic regions of the genome which are rich in transposable elements (Fig. 1B). In addition, several retroelement families and the Mutator DNA elements were significantly enriched for OsSRT1-binding (Table 2, Table 3). Second, *OsSRT1* down-regulation led to increased H3K9ac and expression of many transposable elements (this work and ref 6). Finally, a number of OsSRT1 ChIP-seq peaks overlapped with an inverted repeat DNA sequence motif found in the LTR of the Gypsy subfamily, raising the hypothesis that the motif may represent a DNA target sequence of OsSRT1.

## Supporting Information

Figure S1 Western blot analysis of SRT1 in wild type (MH63) and OsSRT1 RNAi plants. Affinity purified anti-OsSRT1 was used in Western blots to detect OsSRT1 levels. *E. coli* –produced OsSRT1-GST fusion was used as positive controls. Detection of histone H3 by anti H3 was used the loading control.
(PPTX)

Figure S2 H3K9ac and OsSRT1 ChIP-seq mapped reads in wild type (MH63) and OsSRT1 RNAi plants. A. H3K9ac and OsSRT1 ChIP-seq mapped reads in wild type (MH63) and OsSRT1 RNAi plants. B. Normalized strand correlation (NSC) and relative strand correlation (RSC) values of the ChIP-seq reads, calculated according to http://www.ncbi.nlm.nih.gov/pmc/articles/PMC3431496/.
(PPTX)

Figure S3 Gene expression level is positively correlated to H3K9ac. Rice genes were divided into 3 tiers (low, middle and high) according to microarray signals and compared to average H3K9ac sequence tag density.
(PPTX)

Figure S4 Gene ontology analysis of genes with increased H3K9ac (FDR<0.05). The red columns represent *OsSRT1* RNAi plants and the blue columns represent the total genes.
(PPTX)

Figure S5 Gene ontology analysis of OsSRT1-binding genes (FDR<0.05). A. OsSRT1-binding genes (red) compared to the total genomic genes (blue). B. Genes with both OsSRT1-binding and expression altered in RNAis plants.
(PPTX)

Figure S6 Two genomic regions that show increased H3K9ac *in OsSRT1* RNAi plants and OsSRT1-binding.
(PPTX)

Table S1 List of genes that showed OsSRT1 binding and H3K9ac increase in *OsSRT1* RNAi plants. The 37 genes on which OsSRT1-binding peaks overlapped with that of H3K9ac increases are marked in yellow.
(PDF)
