# Supplementary material for: The Rice NAD+-Dependent Histone Deacetylase OsSRT1 Targets Preferentially to Stress- and Metabolism-Related Genes and Transposable Elements
Source: PLoS One. 2013 Jun 25;8(6):e66807. doi: 10.1371/journal.pone.0066807 (PMC3692531; doi:10.1371/journal.pone.0066807)
Supplement: Figure S1 — Western blot analysis of SRT1 in wild type (MH63) and OsSRT1 RNAi plants. Affinity purified anti-OsSRT1 was used in Western blots to detect OsSRT1 levels. E. coli –produced OsSRT1-GST fusion was used as positive controls. Detection of histone H3 by anti H3 was used the loading control. (PPTX) [file pone.0066807.s001.pptx]

## Slide 1
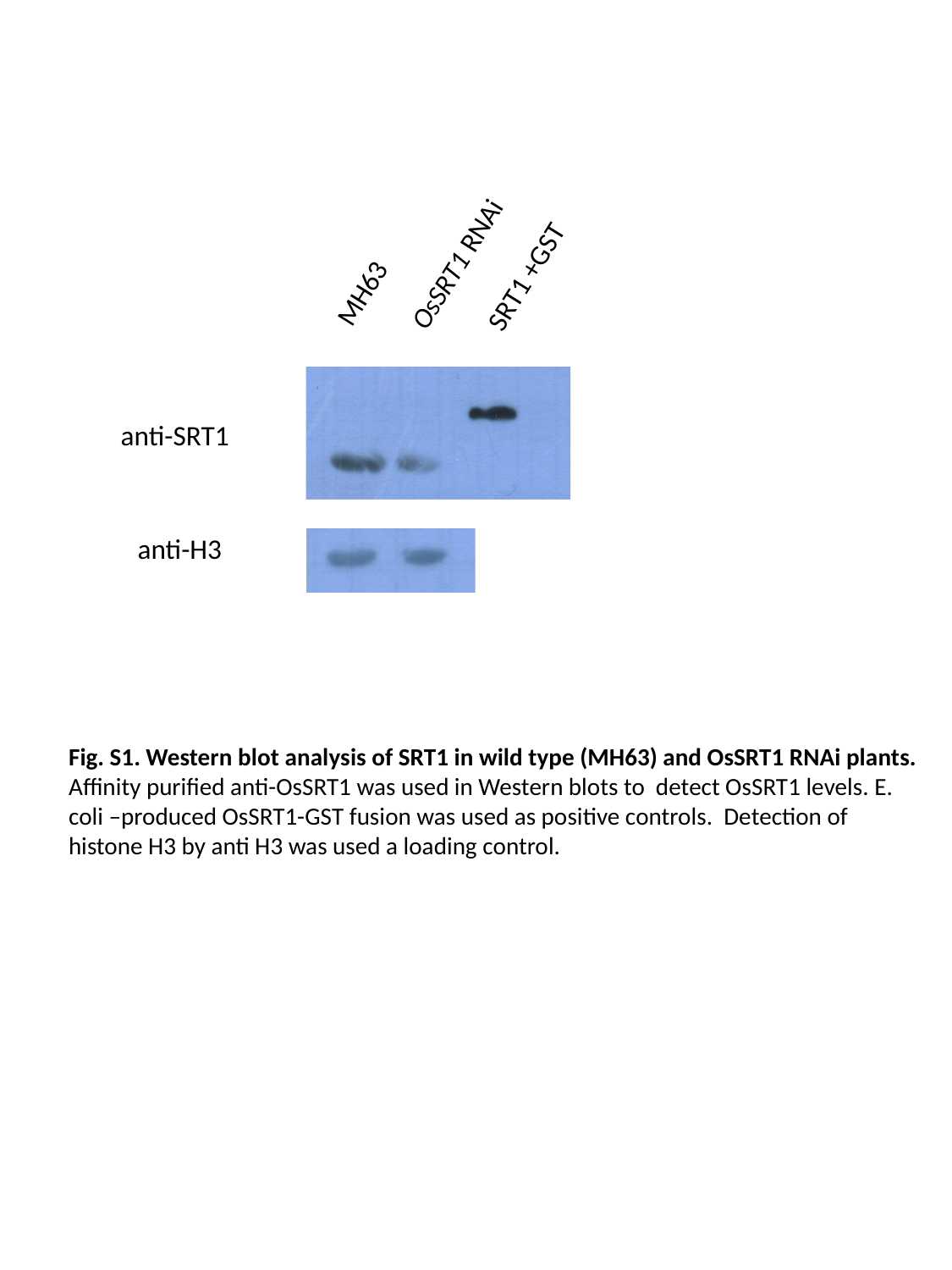

OsSRT1 RNAi
SRT1 +GST
MH63
anti-SRT1
anti-H3
Fig. S1. Western blot analysis of SRT1 in wild type (MH63) and OsSRT1 RNAi plants.
Affinity purified anti-OsSRT1 was used in Western blots to detect OsSRT1 levels. E. coli –produced OsSRT1-GST fusion was used as positive controls. Detection of histone H3 by anti H3 was used a loading control.
