# Supplementary material for: The Rice NAD+-Dependent Histone Deacetylase OsSRT1 Targets Preferentially to Stress- and Metabolism-Related Genes and Transposable Elements
Source: PLoS One. 2013 Jun 25;8(6):e66807. doi: 10.1371/journal.pone.0066807 (PMC3692531; doi:10.1371/journal.pone.0066807)
Supplement: Figure S2 — H3K9ac and OsSRT1 ChIP-seq mapped reads in wild type (MH63) and OsSRT1 RNAi plants. A. H3K9ac and OsSRT1 ChIP-seq mapped reads in wild type (MH63) and OsSRT1 RNAi plants. B. Normalized strand correlation (NSC) and relative strand correlation (RSC) values of the ChIP-seq reads, calculated according to http://www.ncbi.nlm.nih.gov/pmc/articles/PMC3431496/. (PPTX) [file pone.0066807.s002.pptx]

## Slide 1
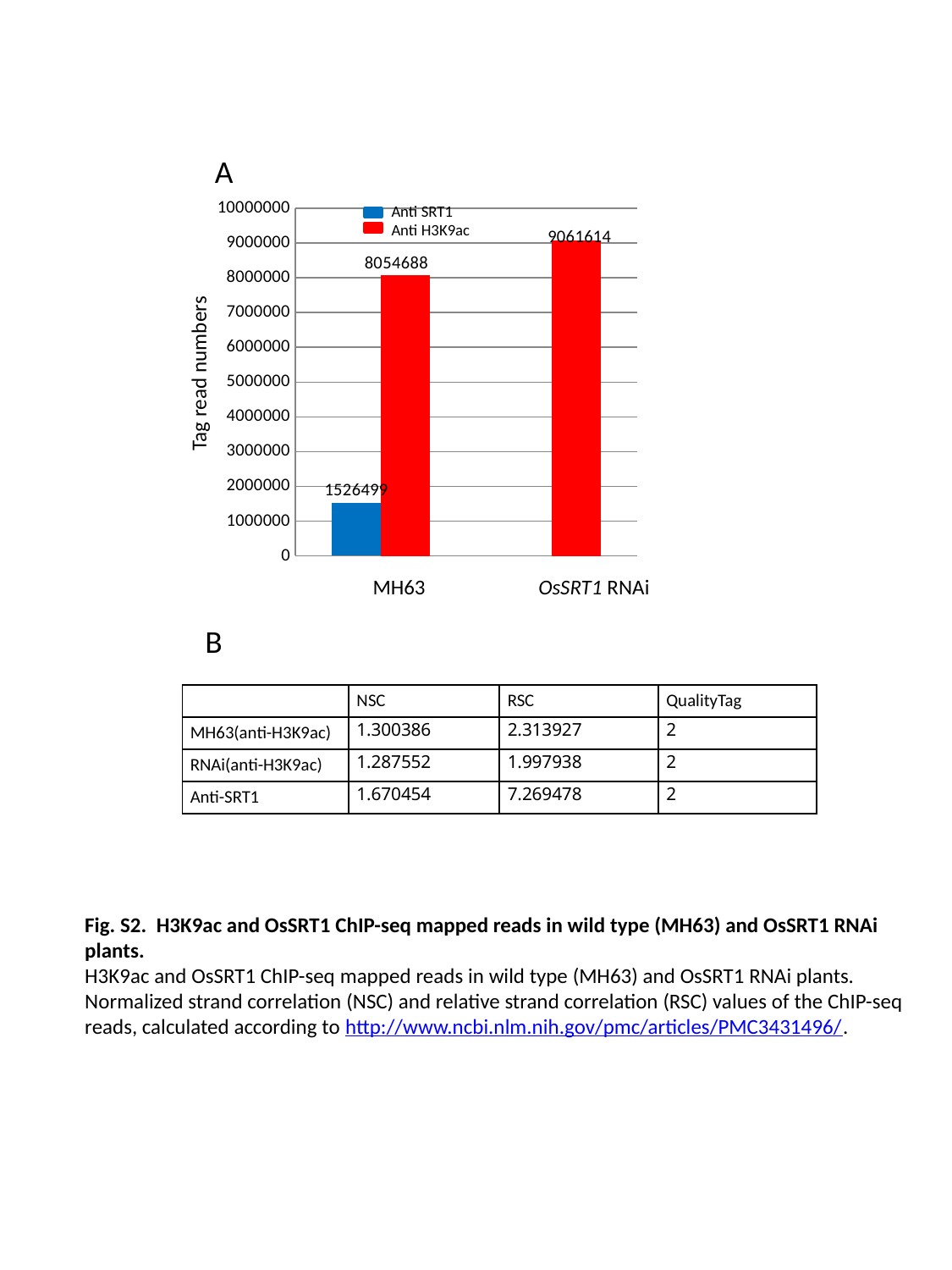

A
### Chart
| Category | anti-SRT1 reads数 | anti-H3K9ac reads数 |
|---|---|---|
| MH63 | 1526499.0 | 8054688.0 |
| srt1-RNAi | None | 9061614.0 |Anti SRT1
Anti H3K9ac
Tag read numbers
MH63 	 OsSRT1 RNAi
B
| | NSC | RSC | QualityTag |
| --- | --- | --- | --- |
| MH63(anti-H3K9ac) | 1.300386 | 2.313927 | 2 |
| RNAi(anti-H3K9ac) | 1.287552 | 1.997938 | 2 |
| Anti-SRT1 | 1.670454 | 7.269478 | 2 |
Fig. S2. H3K9ac and OsSRT1 ChIP-seq mapped reads in wild type (MH63) and OsSRT1 RNAi plants.
H3K9ac and OsSRT1 ChIP-seq mapped reads in wild type (MH63) and OsSRT1 RNAi plants.
Normalized strand correlation (NSC) and relative strand correlation (RSC) values of the ChIP-seq reads, calculated according to http://www.ncbi.nlm.nih.gov/pmc/articles/PMC3431496/.
