# Supplementary material for: The Rice NAD+-Dependent Histone Deacetylase OsSRT1 Targets Preferentially to Stress- and Metabolism-Related Genes and Transposable Elements
Source: PLoS One. 2013 Jun 25;8(6):e66807. doi: 10.1371/journal.pone.0066807 (PMC3692531; doi:10.1371/journal.pone.0066807)
Supplement: Figure S3 — Gene expression level is positively correlated to H3K9ac. Rice genes were divided into 3 tiers (low, middle and high) according to microarray signals and compared to average H3K9ac sequence tag density. (PPTX) [file pone.0066807.s003.pptx]

## Slide 1
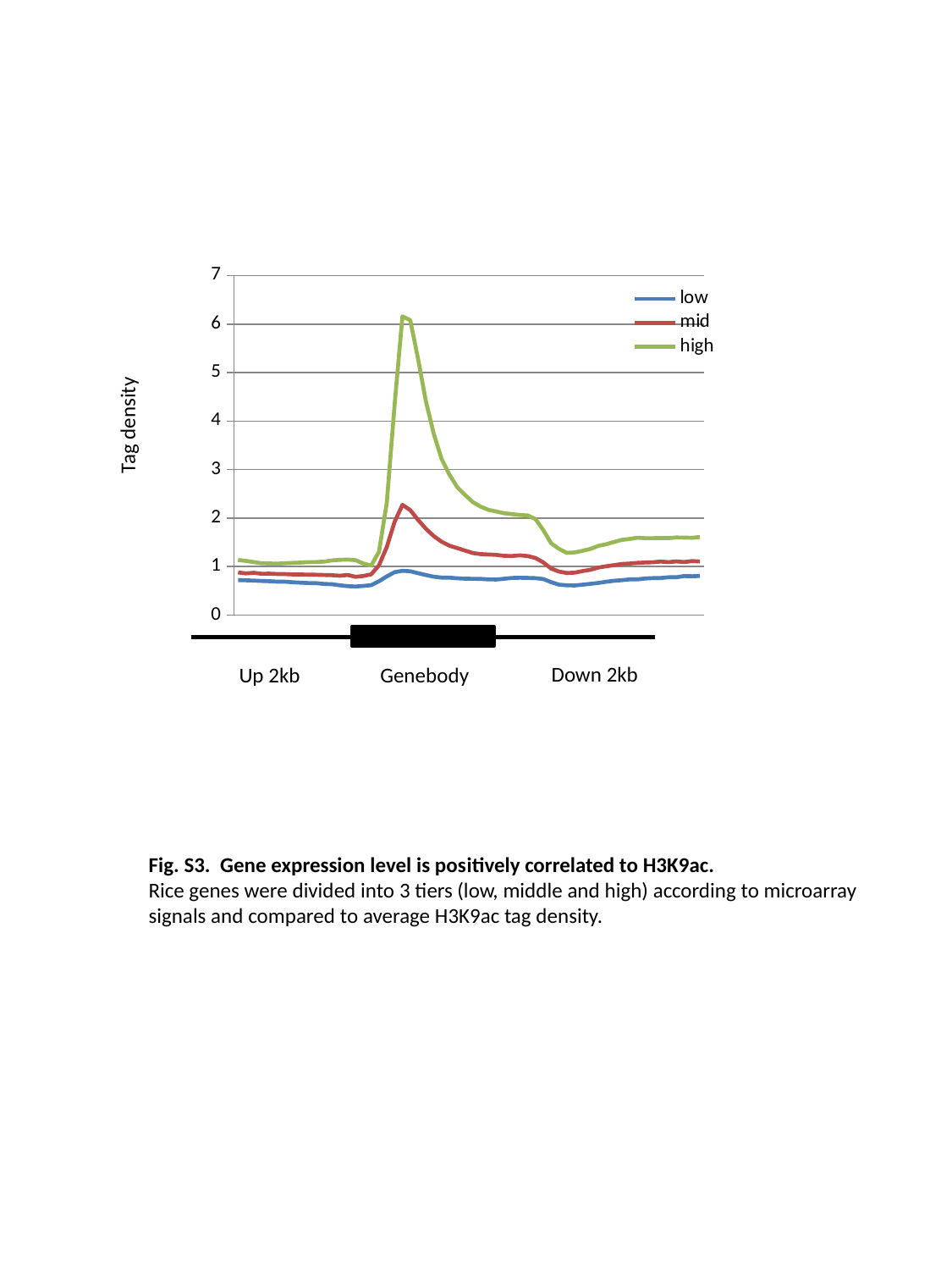

### Chart
| Category | low | mid | high |
|---|---|---|---|
| 2Kb | 0.7217292700212696 | 0.879755083996475 | 1.13663876651983 |
| | 0.717526576895827 | 0.860410256410267 | 1.11804229074891 |
| | 0.7114041459957553 | 0.872212201591524 | 1.0934872246696201 |
| | 0.70458628632177 | 0.8523757736516456 | 1.06811101321587 |
| | 0.6989829907866856 | 0.8551078691423596 | 1.06538942731279 |
| 1.5Kb | 0.690308291991506 | 0.8479832007073506 | 1.0595779735682944 |
| | 0.68848866052446 | 0.845495137046874 | 1.0699859030837144 |
| | 0.6772997873848465 | 0.8387736516357307 | 1.0762017621145499 |
| | 0.6682600992204206 | 0.8382785145888697 | 1.0825850220264401 |
| | 0.6612606307583366 | 0.8343218390804676 | 1.09346872246697 |
| 1Kb | 0.6592124379872495 | 0.832538461538474 | 1.09375859030839 |
| | 0.6419932671864067 | 0.826766578249346 | 1.102807929515435 |
| | 0.6368400070871875 | 0.8228629531388256 | 1.1279841409691698 |
| | 0.614303685329563 | 0.811572060123793 | 1.1387022026431899 |
| | 0.5976461729270115 | 0.8279451812555366 | 1.1455920704845999 |
| 0.5Kb | 0.5899991141034877 | 0.7895888594164585 | 1.133237004405304 |
| | 0.6011321757618815 | 0.806694076038917 | 1.0653859030837152 |
| | 0.617542523033317 | 0.840846153846166 | 1.02023788546257 |
| | 0.698472714386971 | 1.0263757736516501 | 1.30603259911896 |
| | 0.7981422749822925 | 1.40455172413795 | 2.3193339207048367 |
| 0 | 0.886167907904885 | 1.92419349315076 | 4.3454367503061855 |
| | 0.913887431551467 | 2.2731933206961212 | 6.1585250477611755 |
| | 0.904622170318313 | 2.16327566679171 | 6.0781625776312795 |
| | 0.863957042827708 | 1.9603024268231573 | 5.276344420625147 |
| | 0.8264258655900766 | 1.7805290866677699 | 4.406686290613317 |
| 25% | 0.791678759834399 | 1.629021665709576 | 3.73546316634831 |
| | 0.7729563219910316 | 1.5129733848029798 | 3.218750258450326 |
| | 0.7723561321271016 | 1.4306263350882298 | 2.8961919133257013 |
| | 0.7589861535606987 | 1.38115903292694 | 2.6359093908434077 |
| | 0.750341195739552 | 1.330228520384734 | 2.474324520958 |
| 50% | 0.7481266537494536 | 1.2792292943177455 | 2.32851018942044 |
| | 0.7464544005541466 | 1.25721094305198 | 2.23440830075754 |
| | 0.7366274915814118 | 1.2491866823073055 | 2.16863021047616 |
| | 0.7344768459416525 | 1.24111143431732 | 2.1342541195839777 |
| | 0.7494003891326443 | 1.2216139737241098 | 2.099524351824001 |
| 75% | 0.7658667459608025 | 1.2185930670970855 | 2.08065063264175 |
| | 0.770260076079952 | 1.2318853895223199 | 2.06370699257466 |
| | 0.7669695327465912 | 1.2173214320448627 | 2.05382110965659 |
| | 0.762095024956025 | 1.1768462906014 | 1.978775458905715 |
| 100% | 0.743827276909247 | 1.0855675394468647 | 1.751735094837446 |
| | 0.6791805457122715 | 0.9596790450928506 | 1.4804977973568398 |
| | 0.6284514528703182 | 0.897877099911596 | 1.365622026431735 |
| | 0.61518869596032 | 0.868381962864731 | 1.2834502202643199 |
| | 0.6113474486180147 | 0.8757259062776442 | 1.2920440528634498 |
| 0.5Kb | 0.6273130758327518 | 0.9069973474801176 | 1.32444581497798 |
| | 0.6446624734231182 | 0.9339363395225555 | 1.36423876651984 |
| | 0.662646172927015 | 0.9798320070733982 | 1.42652246696036 |
| | 0.687324592487608 | 1.00598496905395 | 1.4591797356828198 |
| | 0.7073414245216266 | 1.02924403183025 | 1.506518942731284 |
| 1Kb | 0.719642097802986 | 1.05338992042441 | 1.550082819383266 |
| | 0.7359390503189376 | 1.063045977011514 | 1.568718061674014 |
| | 0.736189759036155 | 1.07601768346596 | 1.59583259911894 |
| | 0.7553304394046896 | 1.0841980548187544 | 1.585409691629944 |
| | 0.7626736357193576 | 1.08978426171531 | 1.5861462555066 |
| 1.5Kb | 0.7647856130404127 | 1.1046003536693298 | 1.58849955947136 |
| | 0.780643160878819 | 1.0920557029177844 | 1.587093392070472 |
| | 0.7806387313961882 | 1.107002652519896 | 1.601331277533026 |
| | 0.805660878809365 | 1.0936887709991199 | 1.597931277533035 |
| | 0.801380226789519 | 1.11404155614501 | 1.593506607929506 |
| 2Kb | 0.807820694542889 | 1.10514412024758 | 1.6103638766519701 |Tag density
Down 2kb
Up 2kb
Genebody
Fig. S3. Gene expression level is positively correlated to H3K9ac.
Rice genes were divided into 3 tiers (low, middle and high) according to microarray
signals and compared to average H3K9ac tag density.
