# Supplementary material for: The Rice NAD+-Dependent Histone Deacetylase OsSRT1 Targets Preferentially to Stress- and Metabolism-Related Genes and Transposable Elements
Source: PLoS One. 2013 Jun 25;8(6):e66807. doi: 10.1371/journal.pone.0066807 (PMC3692531; doi:10.1371/journal.pone.0066807)
Supplement: Figure S4 — Gene ontology analysis of genes with increased H3K9ac (FDR<0.05). The red columns represent OsSRT1 RNAi plants and the blue columns represent the total genes. (PPTX) [file pone.0066807.s004.pptx]

## Slide 1
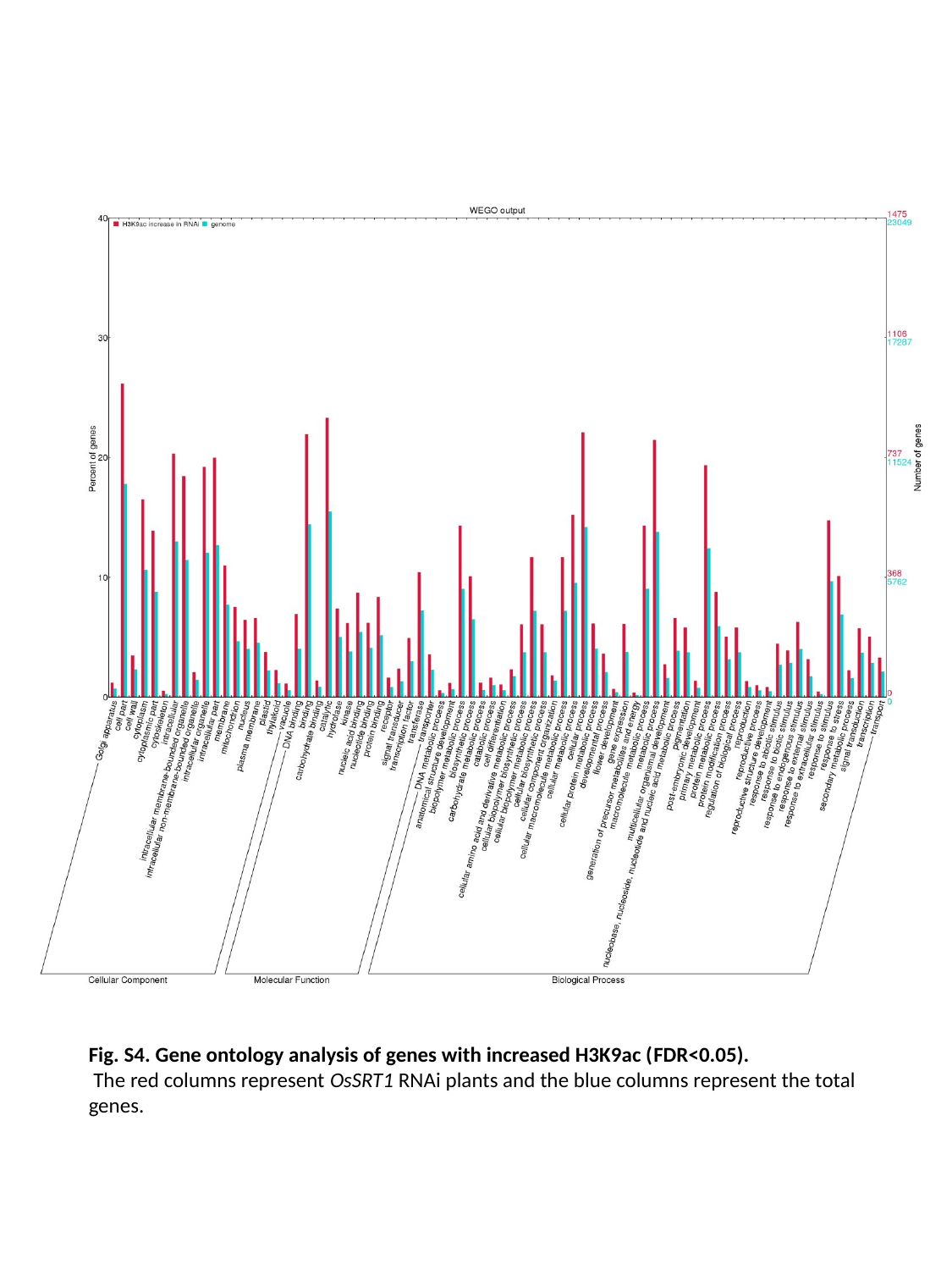

Fig. S4. Gene ontology analysis of genes with increased H3K9ac (FDR<0.05).
 The red columns represent OsSRT1 RNAi plants and the blue columns represent the total genes.
