# Supplementary material for: The Rice NAD+-Dependent Histone Deacetylase OsSRT1 Targets Preferentially to Stress- and Metabolism-Related Genes and Transposable Elements
Source: PLoS One. 2013 Jun 25;8(6):e66807. doi: 10.1371/journal.pone.0066807 (PMC3692531; doi:10.1371/journal.pone.0066807)
Supplement: Figure S5 — Gene ontology analysis of OsSRT1-binding genes (FDR<0.05). A. OsSRT1-binding genes (red) compared to the total genomic genes (blue). B. Genes with both OsSRT1-binding and expression altered in RNAis plants. (PPTX) [file pone.0066807.s005.pptx]

## Slide 1
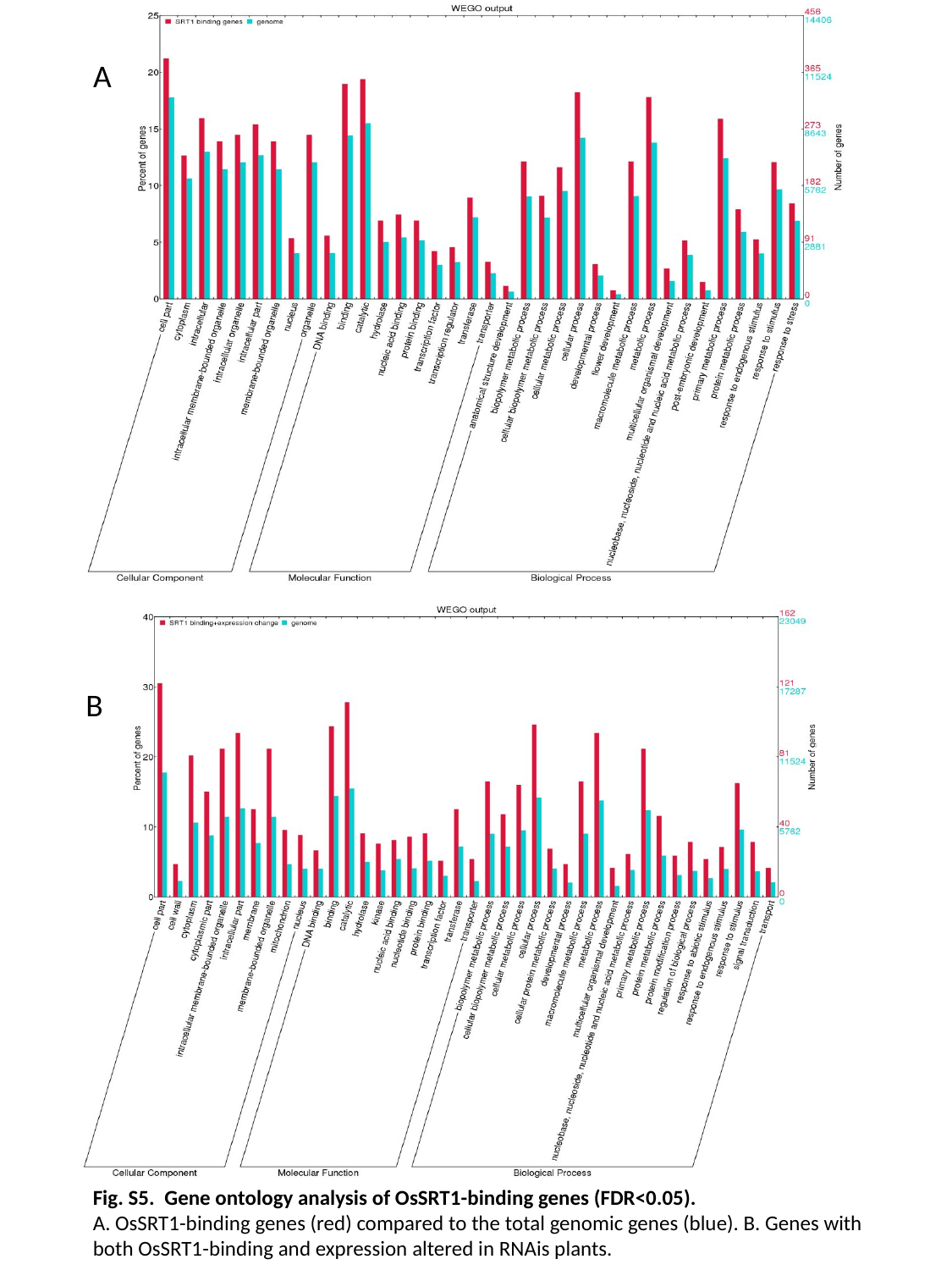

A
B
Fig. S5. Gene ontology analysis of OsSRT1-binding genes (FDR<0.05).
A. OsSRT1-binding genes (red) compared to the total genomic genes (blue). B. Genes with both OsSRT1-binding and expression altered in RNAis plants.
