# Supplementary figures and images for: The Rice NAD+-Dependent Histone Deacetylase OsSRT1 Targets Preferentially to Stress- and Metabolism-Related Genes and Transposable Elements
Source: PLoS One. 2013 Jun 25;8(6):e66807. doi: 10.1371/journal.pone.0066807 (PMC3692531; doi:10.1371/journal.pone.0066807)

## Slide 1
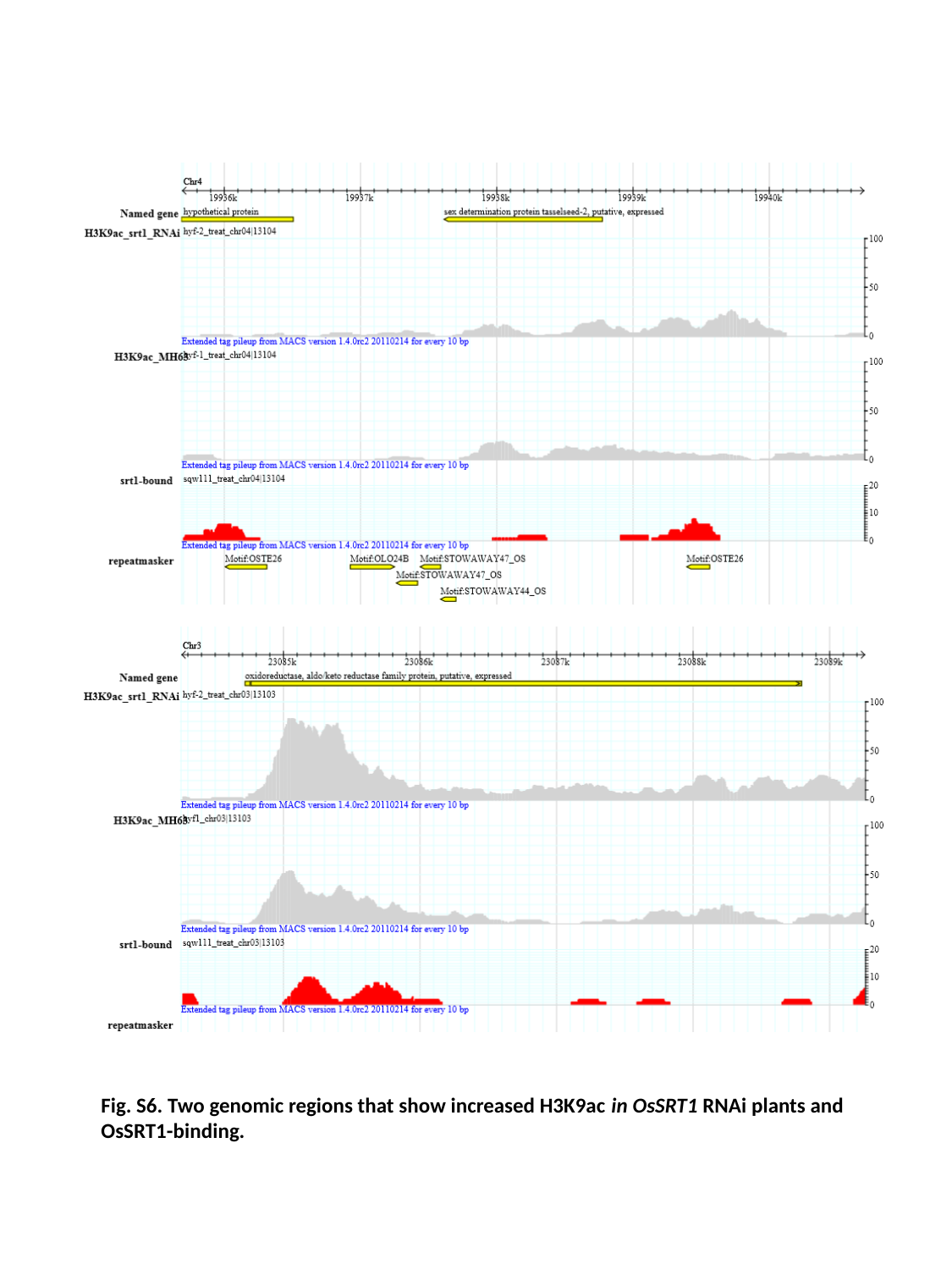

Fig. S6. Two genomic regions that show increased H3K9ac in OsSRT1 RNAi plants and OsSRT1-binding.

Supplement: Figure S6 — Two genomic regions that show increased H3K9ac in OsSRT1 RNAi plants and OsSRT1-binding. (PPTX) [file pone.0066807.s006.pptx]
