# Supplementary material for: The Rice NAD+-Dependent Histone Deacetylase OsSRT1 Targets Preferentially to Stress- and Metabolism-Related Genes and Transposable Elements
Source: PLoS One. 2013 Jun 25;8(6):e66807. doi: 10.1371/journal.pone.0066807 (PMC3692531; doi:10.1371/journal.pone.0066807)
Supplement: Table S1 — List of genes that showed OsSRT1 binding and H3K9ac increase in OsSRT1 RNAi plants. The 37 genes on which OsSRT1-binding peaks overlapped with that of H3K9ac increases are marked in yellow. (PDF) [file pone.0066807.s007.pdf]

Table S1. List of genes that showed OsSRT1 binding and H3K9ac increase in *OsSRT1* RNAi plants. The 37 genes on which OsSRT1-binding peaks overlapped with that of H3K9ac increases are marked in yellow.

MSU Locus	**OsSRT1 ChIP seq reads**	**H3K9ac ChIP-seq reads**		**Gene function Annotation**	**Microarray folds in RNAi versus WT plants**		
		**MH63 (WT)**	**OsSRT1 RNAi**		Repeat1	Repeat 2	Repeat 3
LOC_Os05g41290	33	114	323	disease resistance RPP13-like protein 1, putative, expressed			
LOC_Os08g12780	10	133	273	chloroplast envelope membrane protein, putative, expressed			
LOC_Os07g35335	10	86	234	expressed protein			
LOC_Os01g52304	25	147	485	NB-ARC domain containing protein, expressed			
LOC_Os03g07310	11	120	1246	growth regulator related protein, putative, expressed			
LOC_Os01g05090	11	127	327	expressed protein			
LOC_Os04g42000	14	184	514	6,7-dimethyl-8-ribityllumazine synthase, chloroplast precursor, putative, expressed	1,5157	1,2311	1,516
LOC_Os11g45000	36	49	106	retrotransposon protein, putative, unclassified			
LOC_Os12g41700	11	202	437	LSD1 zinc finger domain containing protein, expressed	1,2311	1,2311	1,32
LOC_Os06g11440	15	41	313	transposon protein, putative, Mutator sub-class, expressed			
LOC_Os01g31580	14	216	466	BZIP protein, putative, expressed			
LOC_Os03g07320	11	120	348	transposon protein, putative, Pong sub-class			
LOC_Os03g41510	24	137	408	oxidoreductase, aldo/keto reductase family protein, putative, expressed			
LOC_Os08g42670	34	136	342	resistance protein, putative, expressed			
LOC_Os03g11910	17	136	329	DnaK family protein, putative, expressed			
LOC_Os11g44990	36	49	106	NB-ARC domain containing protein, expressed			
LOC_Os03g37820	18	44	122	hypothetical protein			
LOC_Os08g15840	14	180	405	ankyrin repeat-rich protein, putative, expressed	1,0718	2	1,072
LOC_Os05g34010	12	51	115	peptide transporter PTR2, putative, expressed			
LOC_Os01g02880	23	173	371	fructose-bisphospate aldolase isozyme, putative, expressed	1,3195	1,0718	1,072
LOC_Os05g03200	12	83	453	expressed protein			
LOC_Os01g18230	17	57	183	FAD-linked oxidoreductase protein, putative, expressed			
LOC_Os04g53680	20	236	580	cyclin, putative, expressed			
LOC_Os03g62160	10	135	334	expressed protein			
LOC_Os08g36790	17	146	306	bZIP transcription factor, putative, expressed			
LOC_Os02g17470	10	72	209	RNA-binding protein-related, putative, expressed			
LOC_Os02g33540	10	54	128	translational activator family protein, putative, expressed			

LOC_Os05g34854	11	55	244	gibberellin 20 oxidase 2, putative, expressed			
LOC_Os07g30170	17	97	240	nitrilase, putative, expressed			
LOC_Os01g12890	20	163	466	expressed protein			
LOC_Os06g45020	10	93	480	STRUBBELIG-RECEPTOR FAMILY 3 precursor, putative, expressed			
LOC_Os03g41339	10	77	258	hypothetical protein			
LOC_Os06g05710	16	268	693	pollen-specific protein, putative, expressed	9,1896	5,6569	3,031
LOC_Os02g03100	14	233	511	oxidoreductase, aldo/keto reductase family protein, putative, expressed	1,0718	1,2311	1,231
LOC_Os03g53020	14	282	574	helix-loop-helix DNA-binding domain containing protein, expressed			
LOC_Os10g42950	16	506	1156	cyclin-dependent kinase E-1, putative, expressed			
LOC_Os02g03090	14	233	511	expressed protein	1,2311	1,4142	1,072
LOC_Os06g51230	10	153	788	conserved hypothetical protein			
LOC_Os12g39420	10		528	nucleobase-ascorbate transporter, putative, expressed	2,2974	1,5157	1,149
LOC_Os07g10550	26		132	cyclin-related protein, putative, expressed			
LOC_Os06g10130	16		224	expressed protein			
LOC_Os02g58170	18		53	transposon protein, putative, unclassified	1,1487	1,4142	8
LOC_Os11g07870	20		187	DEAD/DEAH box helicase domain containing protein, expressed			
LOC_Os08g38850	17		305	phosphatidylinositol transfer, putative, expressed	1,8661	1,2311	1,149
LOC_Os07g37270	10		45	MSP domain containing protein, putative, expressed	1,2311	1,1487	1,231
LOC_Os05g34100	12		127	hypothetical protein			
LOC_Os04g42100	15		624	retrotransposon protein, putative, unclassified, expressed			
LOC_Os06g50280	14		52	OsFBL31 - F-box domain and LRR containing protein			
LOC_Os05g45140	18		36	glucosyl transferase, putative, expressed			
LOC_Os07g47840	17		63	expressed protein			
LOC_Os03g62150	10		54	heat shock protein DnaJ, putative			
LOC_Os11g10710	16		49	protein kinase domain containing protein, expressed	2,1435	1,4142	1,625
LOC_Os10g29650	26		291	retrotransposon protein, putative, unclassified, expressed			
LOC_Os03g07330	11		115	pentatricopeptide, putative, expressed			
LOC_Os01g50420	12		59	STE_MEKK_ste11_MAP3K.7 - STE kinases , expressed			
LOC_Os04g49500	14		221	U-box domain-containing protein, putative, expressed			
LOC_Os02g29190	30		67	ankyrin, putative, expressed	3,249	3,4822	4,925
LOC_Os12g08720	15		65	ramtrack, Broad Complex BTB domain with H family conserved sequence, expressed			

LOC_Os05g28530	10	45	glutaredoxin, putative			
LOC_Os03g25250	12	44	OsFBX90 - F-box domain containing protein			
LOC_Os01g35950	12	39	hypothetical protein			
LOC_Os12g05655	10	459	expressed protein			
LOC_Os04g43560	13	97	no apical meristem protein, putative, expressed			
LOC_Os12g34940	14	61	hypothetical protein			
LOC_Os02g07960	15	261	STRUBBELIG-RECEPTOR FAMILY 3 precursor, putative, expressed	1,5157	1,7411	1,516
LOC_Os04g07830	14	67	conserved hypothetical protein	1,0718	2,1435	1,414
LOC_Os02g07860	15	82	conserved hypothetical protein			
LOC_Os03g03900	12	299	NIN, putative, expressed			
LOC_Os01g65200	10	245	proton-dependent oligopeptide transport, putative, expressed	1,3195	1,4142	1,516
LOC_Os01g66840	10	619	pectinacetylesterase domain containing protein			
LOC_Os06g19170	22	519	cadmium tolerance factor, putative, expressed			
LOC_Os01g50140	11	56	transposon protein, putative, unclassified			
LOC_Os03g45830	32	65	OsSAUR15 - Auxin-responsive SAUR gene family member			
LOC_Os07g35060	10	187	OsFBX238 - F-box domain containing protein, expressed			
LOC_Os04g43700	10	502	glycosyl transferase 8 domain containing protein, putative, expressed			
LOC_Os01g12570	10	78	3-methyl-2-oxobutanoate hydroxymethyltransferase, putative, expressed			
LOC_Os10g43050	12	192	OsClp11 - Putative Clp protease homologue, expressed			
LOC_Os01g13830	14	51	expressed protein			
LOC_Os03g64140	10	67	expressed protein			
LOC_Os12g35760	31	46	transposon protein, putative, unclassified			
LOC_Os03g05820	14	44	kinesin motor domain containing protein, expressed	1,8661	1,1487	1,072
LOC_Os02g51770	28	372	TLD family protein, putative, expressed			
LOC_Os04g46280	10	213	hydrolase, NUDIX family, domain containing protein, expressed			
LOC_Os03g18810	16	203	aminotransferase, classes I and II, domain containing protein, expressed			
LOC_Os12g39410	10	528	hypothetical protein			
LOC_Os06g48500	13	349	expressed protein			
LOC_Os01g65210	10	245	proton-dependent oligopeptide transport, putative, expressed	1,2311	1,4142	1,516
LOC_Os04g55720	19	98	D-3-phosphoglycerate dehydrogenase, chloroplast precursor, putative, expressed			
LOC_Os04g18830	10	140	MATH domain containing protein			

LOC_Os02g14150	12	57	transposon protein, putative, unclassified			
LOC_Os05g27890	12	53	hypothetical protein			
LOC_Os06g23980	14	177	transcription factor, putative			
LOC_Os04g46290	10	213	ethylene-responsive element-binding protein, putative, expressed			
LOC_Os11g45410	10	82	conserved hypothetical protein			
LOC_Os02g56560	14	376	CK1_CaseinKinase_1.6 - CK1 includes the casein kinase 1 kinases, expressed			
LOC_Os05g41160	10	121	expressed protein			
LOC_Os03g19670	12	165	GDSL-like lipase/acylhydrolase, putative, expressed			
LOC_Os12g34950	14	61	retrotransposon protein, putative, unclassified			
LOC_Os02g42950	31	57	YABBY domain containing protein, putative, expressed			
LOC_Os04g32980	14	462	protein binding protein, putative, expressed			
LOC_Os01g04690	10	61	hypothetical protein	2	1,4142	1,741
LOC_Os03g57950	36	222	type I inositol-1,4,5-trisphosphate 5-phosphatase, putative, expressed			
LOC_Os04g57140	20	199	kinesin motor domain containing protein, putative, expressed			
LOC_Os11g23770	13	83	cysteine protease, putative, expressed			
LOC_Os06g24980	11	67	transposon protein, putative, CACTA, En/Spm sub-class			
LOC_Os08g15070	14	59	GDU1, putative			
LOC_Os08g40510	10	533	KID-containing protein, putative, expressed			
LOC_Os06g09540	19	358	SAC domain containing protein, putative, expressed			
LOC_Os06g38480	14	42	retrotransposon protein, putative, unclassified, expressed			
LOC_Os03g19950	10	45	hypothetical protein			
LOC_Os11g38640	12	130	expressed protein			
LOC_Os08g13180	10	40	ramtrack, Broad Complex BTB domain with Meprin and TRAF Homology MATH domain			
LOC_Os01g34080	14	63	armadillo/beta-catenin-like repeat family protein, expressed			
LOC_Os10g33650	16	159	CK1_CaseinKinase_1.9 - CK1 includes the casein kinase 1 kinases, expressed	1,6245	1,0718	1,231
LOC_Os03g11600	35	131	YABBY domain containing protein, putative, expressed			
LOC_Os09g31210	10	63	AGC_PVPK_like_CDK8.1 - ACG kinases include homologs to PKA, PKG and PKC, expressed			
LOC_Os01g04680	10	61	retrotransposon protein, putative, unclassified	1,0718	1,0718	1,072
LOC_Os06g14050	16	80	membrane attack complex component/perforin/complement C9, putative, expressed			
LOC_Os03g20580	10	103	expressed protein			
LOC_Os02g35610	16	171	expressed protein	1,1487	1,0718	1,231

LOC_Os05g37350	10	64	hhH-GPD superfamily base excision DNA repair protein	1,7411	1,8661	1,32
LOC_Os04g32470	11	93	expressed protein			
LOC_Os12g05650	10	459	ACT domain containing protein, expressed			
LOC_Os11g26910	10	173	SKP1-like protein 1B, putative, expressed			
LOC_Os03g25260	12	44	HIT zinc finger domain containing protein, expressed			
LOC_Os07g43370	11	80	amine oxidase family protein, putative, expressed			
LOC_Os04g42770	18	262	expressed protein			
LOC_Os12g37770	10	67	RGH1A, putative, expressed			
LOC_Os06g24990	11	67	xylanase inhibitor protein 1 precursor, putative, expressed	1,2311	1,2311	1,149
LOC_Os03g06530	12	213	hypothetical protein			
LOC_Os12g34470	22	59	hypothetical protein			
LOC_Os02g43380	11	70	hypothetical protein			
LOC_Os10g22430	14	635	gibberellin response modulator protein, putative, expressed			
LOC_Os02g45770	27	66	OsMADS6 - MADS-box family gene with MIKCc type-box, expressed			
LOC_Os04g29350	20	49	retrotransposon protein, putative, unclassified			
LOC_Os12g27220	21	176	transferase family protein, putative, expressed			
LOC_Os03g25280	12	351	peroxidase precursor, putative, expressed			
LOC_Os03g09820	13	40	transcription initiation factor IIB, putative			
LOC_Os07g47300	14	291	spo0B-associated GTP-binding protein, putative, expressed			
LOC_Os02g43390	11	70	retrotransposon protein, putative, Ty3-gypsy subclass			
LOC_Os02g03294	14	330	cyclin, putative, expressed			
LOC_Os01g27260	10	62	glutathione S-transferase, putative, expressed			
LOC_Os10g42230	12	141	AT hook motif domain containing protein, expressed	2,1435	1,6245	1,414
LOC_Os03g25060	12	137	expressed protein			
LOC_Os04g33240	10	61	sex determination protein tasselseed-2, putative, expressed			
LOC_Os06g38490	14	42	retrotransposon protein, putative, unclassified			
LOC_Os01g06450	15	479	glycosyltransferase family 43 protein, putative, expressed			
LOC_Os08g33082	12	196	DNA-repair protein complementing XP-C cells, putative, expressed			
LOC_Os05g41150	10	121	expressed protein			
LOC_Os02g52830	12	69	lipase, putative, expressed			
LOC_Os04g54180	10	307	serine/threonine-protein kinase receptor precursor, putative, expressed			

LOC_Os10g42930	12	475	hypothetical protein			
LOC_Os12g16080	14	87	expressed protein			
LOC_Os06g45080	16	348	rabGAP/TBC domain-containing protein, putative, expressed	1,0718	1,3195	1,072
LOC_Os01g07160	11	77	OsFBX3 - F-box domain containing protein			
LOC_Os01g57420	14	155	diacylglycerol kinase, putative, expressed			
LOC_Os04g37690	18	119	RNA recognition motif containing protein, putative, expressed			
